# Chemical Composition, Intake, and Digestibility of Native and Improved Perennial Grasses and Crop Residues of Burkina Faso

**DOI:** 10.3390/ani16152368

**Published:** 2026-08-03

**Authors:** Gildas M. L. Yoda, Salimata Pousga, Nouhoun Zampaligré, Michel Kéré, Mohamed H. Assouma, Luc H. Dossa

**Affiliations:** 1 Station de Farakoba Institut de l’Environnement et de Recherches Agricoles (INERA)/Centre National de la Recherche Scientifique et Technologique (CNRST), Bobo-Dioulasso 04 BP 8645, Burkina Faso; yodagildas@yahoo.fr; 2 Institut du Développement Rural (IDR), Université Nazi Boni (UNB), Bobo-Dioulasso 01 BP 1091, Burkina Faso; pousgasalimata@yahoo.fr (S.P.); michel_kere@yahoo.fr (M.K.); 3 UMR SELMET, University Montpellier, INRAE, Institut Agro, CIRAD, 34398 Montpellier Cedex 5, France; habibou.assouma@cirad.fr; 4 Laboratoire des Sciences Animales, Faculté des Sciences Agronomiques, University of Abomey-Calavi, Abomey-Calavi 01 BP 526, Benin; dolhip@yahoo.com

**Keywords:** forages, chemical composition, Djallonké sheep, intake and digestibility

## Abstract

In Burkina Faso, native and improved forage species and crop residues are widely used as feed resources for ruminant livestock. Accurate information on their chemical composition, digestibility, and voluntary intake is essential for balanced and cost-effective ration formulation. This study, conducted from 2021 to 2022 at the INERA research station with Djallonké sheep, evaluated the nutritional quality of key forage resources: *Brachiaria* cv. Mulato II, *Panicum maximum* cvs. Zuri and C1, *Andropogon gayanus*, maize and millet straws, and cowpea (*Vigna unguiculata*) and peanut (*Arachis hypogaea*) haulms. Four trials were conducted using six intact adult male sheep per forage. For each trial, the animals were fed ad libitum during a 14-day adaptation period, followed by a 7–8-day measurement phase for intake and digestibility. The chemical composition of each forage was analyzed by NIR spectrometry using ILRI-validated equations for tropical forages and crop residues for Burkina Faso. Protein contents in the hays and straws ranged from 3.81% to 5.72%, with fiber contents of 71.76–78.16%. Forages harvested at heading and preserved as hay had higher nutrient levels than straws, while improved green fodder protein contents exceeded 7%. Peanut (14.75%) and cowpea (14.03%) haulms showed the highest protein concentrations. Intake was greatest for *Panicum maximum* cv. C1 (56.78 g/kg BW^0.75^, *p* < 0.05), with legume haulms (81.10 gDM/kgBW^0.75^) consumed more than green fodder (47.65 gDM/kgBW^0.75^). Digestibility was highest with peanut haulms (62.72%). These findings provide updated insights to strengthen Burkina Faso’s feed database and guide balanced livestock ration formulation.

## Simple Summary

Forage is a key component of ruminant diets. This study evaluates the nutritional quality of the main forage species in Burkina Faso to provide accurate data for cost-effective and balanced ration formulation. Several trials were conducted to assess the proximate composition, intake and digestibility of native and improved forages, including grasses and crop residues. The feed resources assessed included *Brachiaria* cv. Mulato II and *Panicum maximum* cv. Zuri straw, *Andropogon gayanus* hay (native forage), *Panicum maximum* cv. C1 hay, green fodder of *Panicum maximum* sp. and *Brachiaria* cv. Mulato II, and crop residues (maize and millet straw, cowpea and peanut haulms). Nutritional composition varied by species and harvest stage, with improved and native grasses harvested at stem elongation showing higher nutrient contents than cereal straws. Peanut and cowpea haulms contained more protein than green grass fodder. Among hay and straw, *Panicum maximum* cv. C1 was the most ingested feed by *djalonke* sheep, while legume haulms were more readily consumed than green fodder. Digestibility was greatest in peanut haulms. These findings enrich Burkina Faso’s feed database and support more effective livestock ration formulation.

## 1. Introduction

Livestock plays an important socio-economic role in West African countries, particularly in the Sahel [1,2], by providing nutritious food such as meat and milk, incomes, and jobs in addition to its socio-cultural roles. However, the availability of and access to nutritionally adequate feed resources remains a major constraint for ruminant production [3], accounting approximately for 70% of total farm expenses. In Burkina Faso, feed resources include pasture grasses, shrubs, and tree fodder, crop residues such as cereal straws and legume haulms [4,5], agro-industrial by-products like cottonseed cake and cereal brans, and other commercial concentrate feed and ingredients [6]. During the rainy season, green pastures dominate livestock diets, but biomass declines and nutritive value drop sharply at the season’s end [7]. In this context, crop residues then become crucial, either grazed directly or stored for supplementation during the dry season. To address feed shortages, the promotion of cultivated forages and dual-purpose food–feed crops, alongside concentrate feeds, is increasingly encouraged [5].

Cultivated fodder from native and improved forage grasses and legumes is recognized for its high productivity and good feed quality [8]. In Burkina Faso, previous studies have highlighted its potential to improve good-quality feed availability and farmers’ willingness to adopt forage crops as a strategy to mitigate seasonal shortages [4,5]. Effective use of feed resources requires knowledge of their nutritive value, including proximate chemical composition, intake, and digestibility, to guide balanced and cost-effective diet formulation [2]. Beyond ration formulation, forage quality data also support precision feeding and greenhouse gas mitigation through feeding strategies, as ruminants are major enteric methane emitters in pastoral and agro-pastoral production systems [9]. Although several studies have assessed feed composition, intake, and digestibility in West Africa [6,10], updated information is needed for recently introduced forages. This study therefore aimed to provide current and appropriate data on the nutritive value of major forage resources in Burkina Faso.

## 2. Materials and Methods

### 2.1. Study Area

**Figure 1:**
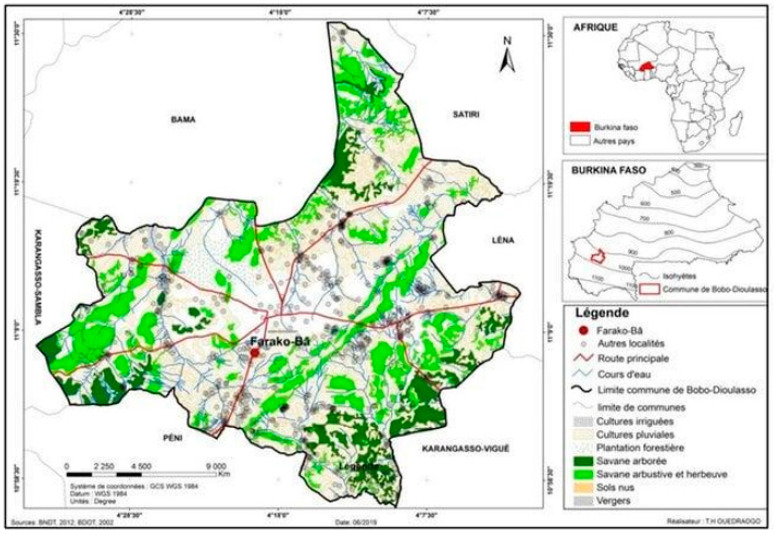
Study site location. Sources: TH OUEDROGO (2019) (Base de données BNDT 2012 et BDOT, (2002).

The experimental trials were conducted at the research station of the Institut de l’Environnement et de Recherches Agricoles (INERA) in Farakoba, on the outskirts of Bobo Dioulasso, Burkina Faso. The site is located at 11°06′ N latitude and 4°20′ W longitude, with a mean elevation of approximately 405 m above sea level (Figure 1). The climate is tropical, with a dry season from November to April (6 to 7 months) and a rainy season from May to October (5 to 6 months). Annual rainfall ranges from 900 to 1300 mm. Temperatures vary between 20 °C (minimum in December) and 35 °C (maximum in April), with an average annual temperature of 27 °C.

### 2.2. Experimental Design and Animals Used in the Trials

Four experimental trials were conducted between 2021 and 2023. Each trial followed a randomized Fisher block design with six uncastrated Djallonké male sheep per forage. All the animals were over 18 months of age and purchased from the local livestock market. The Djallonké breed is the most common sheep breed raised in the study area. Prior to the trials, the animals were dewormed with ivermectin^®^ (Venray, The Netherlands) and supplemented with vitamins for two weeks. Each batch of 6 animals was fed by a single person throughout the trials. For all the trials, water and mineral supplementation were provided ad libitum and no concentrates used.

The first trial, conducted in 2021, evaluated hays from *Brachiaria* cv. Mulato II and *Panicum maximum* cv. Zuri, two perennial hybrid grasses recently introduced in Burkina Faso. Fodder from these species was harvested after fruitification and seed collection.

The second trial, conducted in December 2022, used hay from *Andropogon gayanus* (a native perennial forage collected from fallow land at the Farakoba station) and *Panicum maximum* cv. C1 (an improved hybrid forage cultivated at the station). Both forages were harvested at the haying stage, dried, and stored in a barn.

The third trial evaluated green fodder from *Brachiaria* cv. Mulato II and *Panicum maximum* spp. both cultivated at the Farakoba research station and harvested at the booting stage. Twenty-three (23) elementary plots of 30 m^2^ each were established and were successively harvested at one-day intervals to ensure uniform plant development during the adaptation and measurement phases. The plots covered 15 days of adaptation and 8 days of intake and digestibility measurements. From 60 days of age, the plots were mowed daily to feed the animals. After each harvest, mineral fertilizer (N23P10K05 at a rate of 100 kg/ha) was applied to promote regrowth.

The fourth trial focused on crop residues commonly used during the dry season, including maize and millet straws as well as peanut and cowpea haulms.

### 2.3. Data Collection

For intake and digestibility assessments, the animals were allocated to forage groups according to body weight. All forage types were manually chopped before distribution. The forage samples were offered ad libitum in two daily meals (09:00 and 16:00). Refusal rates varied by ration type. As noted by Kaboré-Zoungrana et al. [10], the 10% refusal rate commonly tolerated for temperate fodder is difficult to apply to tropical fodder due to its coarser nature. Refusal rates of 15–25% have been reported for green fodder and legume haulms and up to 40% for hay [11]. For cereal straw, distribution was fixed at 4% of live weight in dry matter [11].

The animals were fitted with fecal collection bags and housed in digestibility cages equipped with feeders and drinking troughs. At the beginning and end of each period, the animals were weighed to monitor body weight variation and adjust fodder quantities accordingly. Each trial included a 2-week phase of adaptation to the fecal collection bags and experimental diets, followed by a 7–8-day data collection period during which daily feces and refusals were collected. For all four trials, samples of the distributed fodder, feces and refusals were collected daily for laboratory analyses. The samples collected were air-dried, oven-dried at 65 °C for 48 h and grounded 1 mm prior to analyses.

### 2.4. Proximate Composition Analyses

The chemical composition of forage and feces was determined at the laboratory of the Institut de l’Environnement et de recherche Agricoles (INERA). The analyses included dry matter (DM) content, mineral matter (MM) or total ash, organic matter (OM), crude protein (CP), and cell wall components: neutral detergent fiber (NDF), acid detergent fiber (ADF), and acid detergent lignin (ADL). These analyses were performed using near-infrared spectroscopy (NIRS DS 2500 Hillerød, Denmark) over the wavelength range 1100–2500 nm. Prediction equations developed by the International Livestock Research Institute (ILRI) were applied for calibration and analysis (ILRI).

### 2.5. Intake and In Vivo Digestibility Measurements

Feed intake was defined as the quantity of feed consumed by each animal over a 24 h period, calculated as the difference between the amount of feed offered on day D and the refusals collected on day D + 1. This calculation was performed individually for each animal and forage type. For each forage studied, the in vivo digestibility of dry matter (DMD) and organic matter (OMD) was calculated using the following formula:

Digestibility (%) = ((Nutrient intake − Nutrient in feces)/Nutrient intake) × 100

where Nutrient intake = amount of a specific nutrient (e.g., dry matter, organic matter) consumed by the animal. Nutrient in feces = amount of the same nutrient excreted in the feces.

### 2.6. Data Statistical Analyses

The collected data were recorded using Microsoft Excel 2019 and analyzed using R software (version 4.4).

For the proximate chemical composition of feeds, only descriptive statistics were performed to characterize the nutrient concentrations of each feed resource.

Feed intake data were analyzed using a Student’s *t*-test for Trials 1, 2, and 3, and a one-way analysis of variance (ANOVA) for Trial 4. Mean comparisons were performed using Tukey’s Honestly Significant Difference (HSD) test to identify differences among the feed resources tested in Trial 4. For Trials 1, 2, and 3, where only two feed types were evaluated under the same experimental conditions, the differences between means were assessed using a Student’s *t*-test. Digestibility data were analyzed using the same statistical procedures as those applied to feed intake. Differences were considered statistically significant at *p* < 0.05.

## 3. Results

### 3.1. Forage Chemical Composition

The chemical composition of the forages used in the trials is presented in Table 1. Clear differences were observed between forage groups depending on the physiological stages at harvest. Freshly harvested improved forages had the lowest dry matter content (20–25%). The highest dry matter values were recorded in forages collected after seed harvesting at the end of the rainy season (November and December). Protein content was lowest in hays and cereal straws (3.80–5.77%), while freshly cut cultivated forages showed higher levels (7.07–8.56%). Legume haulms had the highest protein content, with groundnut and cowpea haulm reaching 14.75% and 14.03%, respectively.

Forage grasses, regardless of condition or physiological stage, exhibited high NDF contents. Values ranged from 63.53% to 77.89% in fresh forages and from 71.76% to 78.16% in hays from improved and native forages as well as cereal straws. In contrast, legume haulms had lower NDF concentrations, with the lowest value observed in peanut haulms (45.4%). Peanut haulms also showed higher ME compared with hay and fresh improved fodder harvested at the booting stage.

When comparing offered and refused feed, differences were greater for fresh forage harvested at the haying stage than for hay or straw. This phenomenon is often attributed to the higher moisture content of fresh forage compared to hay. Fresh forage contains substantially more water than hay, which can limit intake due to rumen fill. In addition, it promotes selective feeding behavior in animals, leading to the rejection of less palatable and more fibrous plant components. The results in Table 2 show that fresh offered forages have lower dry matter content (20–25%) than for the hay/straw (79–93%) tested in this study.

### 3.2. Feed Intake of the Improved and Native Perennial Grasses and the Crop Residues Tested

Intake values for the improved and native perennial forage resources are presented by trial in Table 2.

In Trial 1, significant differences (*p* < 0.05) were observed between the two grasses. *Panicum maximum* cv. Zuri showed higher values for dry matter, organic matter, fiber fractions, and energy concentrations, but lower crude protein content compared to *Brachiaria* cv. Mulato II (Table 2).

In Trial 2, a similar trend was observed. *Panicum maximum* cv. C1 exhibited lower crude protein content but higher values for the other parameters compared to the native *Andropogon gayanus* Kunth.

In Trial 3, fresh fodder of *Brachiaria* cv. Mulato II had higher crude protein content and lower acid detergent lignin (ADL) and acid detergent fiber (ADF) concentrations than fresh fodder of *Panicum maximum* spp. However, no significant differences were observed for dry matter, organic matter, or neutral detergent fiber (NDF).

Among the crop residues evaluated in Trial 4, peanut haulms exhibited the highest intake values across all measured parameters, followed by cowpea haulms (Table 3). As expected, legume haulms showed higher values than cereal straws. Among the cereal residues, millet straw displayed higher crude protein intake than maize straw, while no significant differences were observed for the other parameters.

### 3.3. In Vivo Digestibility of Dry and Organic Matter

Regarding the digestibility of the tested feed resources, the results presented in Table 4 indicate that no significant differences were observed in Trials 1, 2, and 3 in terms of dry matter digestibility. A similar pattern was observed for organic matter digestibility in these trials, except in Trial 3, where *Brachiaria* cv. Mulato II showed a higher digestibility value than *Panicum maximum* spp.

In Trial 4, cowpea haulms exhibited the highest digestibility values for both dry matter and organic matter. However, no significant differences for the same parameters were observed among the other feed resources evaluated in this trial (Table 4).

## 4. Discussion

### 4.1. Proximate Composition of the Feed Resources

The results of the chemical composition of the feed resources tested across the trials revealed significant differences in nutrient content depending on harvest stage and forage type. The hays from native and improved perennial forage grasses, as well as cereal straws, had lower nutrient contents than legume haulms and freshly cut perennial grasses. The protein content of the hays and straws was below 7%, indicating low quality. These low values can be attributed to advanced harvest stages and prolonged storage before use. The lower protein levels observed in *Panicum* cultivars compared with other perennial species may be explained by the rapid decline in chemical composition, particularly protein, associated with plant development [12]. Nutritive values decrease during plant growth [13], and this decline is more pronounced under rapid growth [14]. Upright grasses such as *Panicum maximum* cv. C1, which grow rapidly under irrigation, fertilization, and high temperatures, develop faster than spreading grasses (e.g., *Brachiaria* sp.), which grow more slowly, producing 30–40 kg of dry matter per hectare per day, depending on conditions [15]. The crude protein content of *Brachiaria* cv. Mulato II is comparable to values reported by Zampaligré et al. [5], whereas *Panicum maximum* cv. C1 had lower protein content (4.1%). The cultivars of perennial species harvested at 60 days of regrowth generally had protein contents equal to or higher than 7%, making them of better quality than cereal straws commonly used in the study area. At this stage, however, they also exhibited high fiber content, like those of cereal straws. The values obtained differ from those reported by Adnew et al. [16] for *Brachiaria* cv. Mulato II, which showed a protein content of 18.76% at 60 days of mowing. This species is widely used in some countries due to its high forage quality [17,18].

As expected, nitrogen concentrations in peanut and cowpea haulms were higher than in cereal straws, which contained elevated levels of parietal constituents. The cereal straws harvested at the end of the rainy season, after cereal grain collection, had particularly high fiber concentration. Despite their low nutritive value, these resources remain an important component of ruminant diets during the dry season in the agro-pastoral systems of the study area. These findings are consistent with those of Nantoumé et al. [19], who reported crude fiber contents of 41.5% for sorghum, 41.8% for maize, and 37.5% for cowpea. Cowpea haulms exhibited higher fiber content (NDF and ADF) than groundnut haulms. The composition of cowpea haulms presented values comparable to those reported for new dual-purpose varieties by Sanfo et al. [20]. The metabolizable energy values obtained for millet were like those of 6.56 MJ/kg reported by Belem et al. [21], while those for maize (7.10 MJ/kg) were slightly lower. The ME values for cowpea were consistent with those (7.7 MJ/kg DM) reported by Sanfo et al. [20].

### 4.2. Feed Intake and Digestibility

Significant differences in dry matter intake (DMI) were observed among the feed resources evaluated, particularly between perennial grasses and cereal straws. Early harvested hays (booting stage) had the highest intake values compared with those harvested after fruiting and grain collection, confirming that late harvesting is associated with reduced intake [10]. The values obtained fall within the range reported by Babatoundé et al. [12] for forage at the late flowering stage (29.7 and 54 g DM/kg BW^0.75^). Intake of *Andropogon gayanus* hay was higher than that reported by Kaboré-Zoungrana et al. [10] at the heading and fruiting stages, with a refusal rate of 31% (38 g/kg BW^0.75^). Differences between forages harvested at the same physiological stage may be linked to variations in stem/leaf ratio, chemical composition (sugar content, fiber degradation rate) [22], and structural characteristics of leaf surfaces, all of which can influence ruminant preference and intake [23,24]. Among the hays tested, *Panicum maximum* cv. C1 was the most consumed by sheep, corroborating the findings of Babatoundé et al. [12], who reported higher intake values for *Panicum* varieties compared with *Brachiaria* varieties. Cereal residues showed low intake values as they have a high refusal rate. Forages with low protein content and high fiber content (NDF and ADF) generally have low intake [25]. Nantoumé et al. [19] reported values of 40 g DM/kg BW^0.75^ for maize straw and 35 g DM/kg BW^0.75^ for millet straw. These results confirm that cereal straws are poor-quality fodders and require supplementation for optimal utilization by ruminants.

Green fodder biomass from improved grasses harvested at the growing stage had lower intake values than legume haulms. This difference may be explained by the superior nutrient composition of legumes compared with grasses, even when harvested green. Groundnut haulms were the most consumed by sheep among the resources tested. Their high nitrogen content contributed to the elevated intake, whereas grasses, characterized by high parietal constituents and low protein content, were less ingested A crude protein content below 7% is generally considered a limiting factor for the intake of poor-quality forages [26].

The values obtained in this study are consistent with those reported by Nantoumé et al. [19], who found higher intake with legume haulms compared with grasses, with groundnut haulms reaching 90 gMS/kg BW^0.75^. Groundnut dry matter intake was significantly higher than that of cowpea (*p* < 0.05), a difference attributable to variations in parietal structures, particularly NDF content. The higher protein intake observed in groundnut haulms suggests that their protein is more readily available than that of cowpea’s. Protein intake values exceeded those reported by Etela and Dung [27] for six groundnut varieties (5.8–7.0 g/kg BW^0.75^). The analysis of refusals indicated that hay and straw intake was influenced by sheep selectivity during feeding. Indeed, sheep tend to consume the more palatable and digestible parts of forage first, such as leaves, before moving to less digestible fractions. This selective behavior has been documented by several authors, including Mero and Udén [28], Kabore-Zoungrana et al. [10], and Babatoundé et al. [12].

In this study, the nitrogen content of hay from perennial grasses and cereal crop straws was below the minimum threshold of 7% DM required for optimal rumen microbial activity [12,29]. Efficient utilization of straw depends on adequate rumen cellulolysis, which requires sufficient nitrogen, minerals, and vitamins, nutrients often deficient in cereal residues [30]. The in vivo digestibility coefficients obtained are within the range reported by Kaboré-Zoungrana et al. [10] for grass hay (30–67% DM). The in vitro organic matter digestibility values are lower for maize straw compared with those reported by Belem et al. [21], but higher for millet straw, with values of 44.8% and 41.83%, respectively, for the two cereal residues.

Differences in forage intake made it difficult to compare digestive utilization coefficients across feed resources, as intake strongly influences passage rate and, consequently, digestibility [28]. One of the main challenges in evaluating the intake and digestibility of tropical feed resources compared with temperate ones is the higher refusal rates, which complicate accurate measurement and increase sorting behavior. The use of NIRS offers an alternative approach, allowing estimation of these values without relying on live animals, which may suffer health problems due to deficiencies in the tested resources.

## 5. Conclusions

This study provides valuable insights into the chemical composition, intake, and digestibility of the major forage resources in Burkina Faso. Hays from native and improved forage grasses generally had higher nutrient concentrations than cereal straws, while improved forage crops harvested at the end of the rainy season exhibited nutrient levels comparable to native grasses. Green fodder from native and improved perennial grasses harvested at the growing stage contained more protein than hay produced after fructification and seed harvest. Among the legume haulms, groundnut haulms were more consumed than cowpea haulms, reflecting their superior nitrogen and protein content. Overall, the haulms commonly used by farmers have higher protein and energy values, along with good digestibility, making them valuable supplements during periods when cereal straw dominate livestock diets. Further research is recommended to determine optimal mowing periods for cultivated improved forage grasses to maximize their nutrient content. The updated nutritional values presented here are crucial for formulating balanced rations and enhancing livestock productivity in Burkina Faso’s agro-pastoral systems, as well as in other West African countries with comparable agro-ecological conditions.

## Figures and Tables

**Table 1:** Proximate chemical composition of forages assessed.

Forage Species	Category	Percentage of DM (%)					MJ/kg DM
		DM	CP	NDF	ADF	ADL	ME
Green fodder harvested at booting stage							
*Brachiaria* cv. Mulato II	Offered	24.34	8.56	63.53	37.75	2.48	7.4
	Refused	29.75	4.01	72.18	48.54	4.01	6.6
*Panicum maximum* spp.	Offered	20.33	7.07	72.04	49.11	4.62	6.7
	Refused	22.01	3.80	77.89	55.44	5.70	6.1
Hay harvested at late fructification and after seed collection							
*Andropogon gayanus*	Offered	91.14	5.72	77.13	47.94	5.78	6.2
	Refused	91.77	4.96	77.28	49.47	5.81	6.2
*Brachiaria* cv. Mulato II	Offered	83.31	5.35	71.76	44.25	5.04	7.0
	Refused	77.27	2.06	77.30	51.27	6.63	6.9
*Panicum maximum* cv. C1	Offered	90.45	3.88	76.52	50.16	5.39	6.5
	Refused	91.10	3.76	78.16	52.54	5.52	6.3
*Panicum maximum* cv. Zuri	Offered	83.68	3.81	74.58	49.35	5.86	7.0
	Refused	79.28	2.25	78.07	53.31	6.68	6.5
Cereal straws and legume haulms harvested after seed collection							
*Maize* (*Zea mays*)	Offered	92.22	3.8	74.22	47.96	5.44	6.3
	Refused	89.97	3.38	75.56	50.44	5.70	6.2
*Millet straw* (*Pennisetum glaucum*)	Offered	92.30	4.52	72.97	48.32	6.20	6.6
	Refused	88.61	3.82	72.67	49.61	6.47	6.7
*Peanut* (*Arachis hypogea*)	Offered	73.20	14.75	45.40	36.17	6.48	8.1
	Refused	72.83	13.35	42.09	43.11	6.59	7.7
*Cowpea* (*Vigna unguiculata*)	Offered	79.00	14.03	50.79	36.96	6.76	8.1
	Refused	82.55	10.24	62.21	54.87	9.72	7.0

DM: Dry Matter; CP: crude protein; ADF: acid detergent fiber; ADL: acid detergent lignin; NDF: neutral detergent fiber; ME: metabolizable energy.

**Table 2:** Dry matter and nutrient intake (g/kg BW^0.75^) of the improved and native perennial grasses evaluated.

Trials Conducted	Trial 1		Trial 2		Trial 3	
Parameters	*Brachiaria* cv. Mulato II	*Panicum maximum* cv. Zuri	*Andropogon gayanus*	*Panicum maximum* cv. C1	*Brachiaria* cv. Mulato II	*Panicum* *maximum* spp.
Dry Matter	39.31 ^b^	44.83 ^a^	48.15 ^a^	56.78 ^b^	47.65 ^a^	45.56 ^a^
Organic Matter	34.86 ^b^	40.85 ^a^	44.39 ^a^	51.59 ^b^	40.16 ^a^	39.36 ^a^
Crude Protein	2.71 ^b^	2.02 b ^a^	2.94 ^a^	2.23 ^b^	4.83 ^a^	3.69 ^b^
NDF	27.18 ^b^	32.73 ^a^	37.20 ^a^	43.14 ^b^	28.76 ^a^	32.03 ^a^
ADF	16.09 ^b^	21.34 ^a^	22.74 ^a^	28.01 ^b^	16.17 ^a^	21.50 ^b^
ADL	1.68 ^b^	2.46 ^a^	2.78 ^a^	3.04 ^b^	0.94 ^a^	1.95 ^b^
Metabolizable Energy (MJ/kg BW^0.75^)	0.27 ^b^	0.32 ^a^	0.30 ^a^	0.36 ^b^	0.37 ^a^	0.32 ^b^

Kg BW^0.75^: Kilogram of metabolic weight; ADF: acid detergent fiber; ADL: acid detergent lignin; NDF: neutral detergent fiber. ^a, b^ Values in the same row followed by the same letters do not differ significantly at the 5% level.

**Table 3:** Intake (g/kg BW^0.75^) of crop residues evaluated.

Parameters	Maize Straw	Millet Straw	Peanut Haulms	Cowpea Haulms
Dry Matter	39.88 ^b^	40.68 ^b^	81.10 ^a^	53.99 ^c^
Organic Matter	37.14 ^b^	37.65 ^b^	71.15 ^a^	47.28 ^c^
Crude Protein	1.71 ^b^	2.18 ^c^	12.56 ^a^	8.74 ^d^
NDF	28.95 ^b^	29.84 ^b^	38.01 ^a^	23.94 ^c^
ADF	17.93 ^bc^	19.05 ^b^	27.09 ^a^	14.63 ^c^
ADL	2.04 ^b^	2.39 ^bc^	5.28 ^a^	2.76 ^c^
Metabolizable Energy (MJ/kg BW^0.75^)	0.26 ^b^	0.26 ^b^	0.89 ^c^	0.67 ^b^

Kg BW^0.75^: Kilogram of metabolic weight; ADF: acid detergent fiber; ADL: acid detergent lignin; NDF: neutral detergent fiber. ^a, b, c, d^: Values in the same row followed by the same letters do not differ significantly at the 5% level.

**Table 4:** Digestibility of the feed resources tested on Djallonké sheep.

Trials Conducted	Feed Resources	DMD (%)	OMD (%)
Trial 1	*Brachiaria* cv. Mulato II	54.77 ^a^	54.87 ^a^
	*Panicum maximum* cv. Zuri	49.88 ^a^	51.71 ^a^
Trial 2	*Panicum maximum* cv. C1	48.81 ^a^	49.50 ^a^
	*Andropogon gayanus*	55.17 ^a^	55.27 ^a^
Trial 3	*Brachiaria* cv. Mulato II	52.12 ^a^	52.31 ^a^
	*Panicum maximum* spp.	46.57 ^a^	47.64 ^b^
Trial 4	Peanut haulms (*Arachis hypogea)*	59.63 ^a^	62.72 ^a^
	Cowpea haulms *(Vigna unguiculata)*	50.06 ^b^	51.58 ^b^
	Maize straw *(Zea mays)*	47.80 ^a^	51.51 ^a^
	Millet straw *(Pennisetum glaucum)*	51.18 ^a^	52.03 ^a^

DMD: Dry matter digestibility; OMD: organic matter digestibility; ^a, b^: values in the same column followed by the same letters do not differ significantly at the 5% level.

## Data Availability

The datasets from the current study are available from the corresponding author upon reasonable request.

## References

[1] Adams F., Ohene-Yankyera K., Aidoo R., Wongnaa C.A. (2021). Economic Benefits of Livestock Management in Ghana. Agric. Econ..

[2] Boote K.J., Adesogan A.T., Balehegn M., Duncan A., Muir J.P., Dubeux J.C.B., Rios E.F. (2022). Fodder Development in Sub-Saharan Africa: An Introduction. Agron. J..

[3] Balehegn M., Duncan A., Tolera A., Ayantunde A.A., Issa S., Karimou M., Zampaligré N., André K., Gnanda I., Varijakshapanicker P. (2020). Improving Adoption of Technologies and Interventions for Increasing Supply of Quality Livestock Feed in Low- and Middle-Income Countries. Glob. Food Secur..

[4] Ouédraogo A., Zampaligré N., Mulubrhan B., Adesogan A.T. (2022). Assessment of Peri-Urban Livestock Producers’ Willingness to Pay for Improved Forages as Cash Crops. Agron. J..

[5] Zampaligré N., Yoda G., Delma J., Sanfo A., Balehegn M., Rios E., Dubeux J.C., Boote K., Adesogan A.T. (2022). Fodder Biomass, Nutritive Value, and Grain Yield of Dual-Purpose Improved Cereal Crops in Burkina Faso. Agron. J..

[6] Amole T., Augustine A., Balehegn M., Adesogan A.T. (2022). Livestock Feed Resources in the West African Sahel. Agron. J..

[7] Zampaligré N., Dossa L.H., Schlecht E. (2013). Contribution of Browse to Ruminant Nutrition across Three Agro-Ecological Zones of Burkina Faso. J. Arid. Environ..

[8] Mutimura M., Ghimire S., Luetz J.M., Ayal D. (2021). *Brachiaria* Grass for Sustainable Livestock Production in Rwanda under Climate Change. Handbook of Climate Change Management: Research, Leadership, Transformation.

[9] Ndao S. (2020). La Contribution de l’Élevage dans les Émissions de Gaz à Effet de Serre des Terroirs Agro-Sylvo-Pastoraux de la Haute Casamance au Sénégal. Ph.D. Thesis.

[10] Zoungrana C.K., Toguyeni A., Sana Y. (1999). Ingestibilité et digestibilité chez le mouton des foins de cinq graminées tropicales. Rev. Elev. Med. Vet. Pays Trop..

[11] Kiema A., Nianogo A.J., Ouédraogo T., Somda J. (2008). Valorisation des ressources alimentaires locales dans l’embouche ovine paysanne: Performances technico-économiques et options de diffusion. Cah. Agric..

[12] Babatoundé S., Oumorou M., Tchabi V.I., Lecomte T., Houinato M., Adandedjan C. (2010). Ingestion volontaire et préférences alimentaires chez des moutons Djallonké nourris avec des graminées et des légumineuses fourragères tropicales cultivées au Bénin. Int. J. Biol. Chem. Sci..

[13] Capstaff N.M., Miller A.J. (2018). Improving the Yield and Nutritional Quality of Forage Crops. Front. Plant Sci..

[14] Onjai-uea N., Paengkoum S., Taethaisong N., Thongpea S., Sinpru B., Surakhunthod J., Meethip W., Purba R.A.P., Paengkoum P. (2022). Effect of Cultivar, Plant Spacing and Harvesting Age on Yield, Characteristics, Chemical Composition, and Anthocyanin Composition of Purple Napier Grass. Animals.

[15] Habermann E., Dias de Oliveira E.A., Contin D.R., Delvecchio G., Viciedo D.O., de Moraes M.A., de Mello Prado R., de Pinho Costa K.A., Braga M.R., Martinez C.A. (2019). Warming and Water Deficit Impact Leaf Photosynthesis and Decrease Forage Quality and Digestibility of a C4 Tropical Grass. Physiol. Plant..

[16] Adnew W., Tsegay B.A., Tassew A., Asmare B. (2019). Effect of Harvesting Stage and Altitude on Agronomic and Qualities of Six *Brachiaria* Grass in Northwestern Ethiopia. Agrolife Sci. J..

[17] Jank L., Barrios S.C., do Valle C.B., Simeão R.M., Alves G.F. (2014). The Value of Improved Pastures to Brazilian Beef Production. Crop Pasture Sci..

[18] Maass B.L., Midega C.A.O., Mutimura M., Rahetlah V.B., Salgado P., Kabirizi J.M., Khan Z.R., Ghimire S.R., Rao I.M. (2015). Homecoming of *Brachiaria*: Improved Hybrids Prove Useful for African Animal Agriculture. East Afr. Agric. For. J..

[19] Nantoumé H., Kouriba A., Togola D., Ouologuem B. (2000). Mesure de la valeur alimentaire de fourrages et de sous-produits utilisés dans l’alimentation des petits ruminants. Rev. Elev. Med. Vet. Pays Trop..

[20] Sanfo A., Zampaligré N., Kulo A.E., Somé S., Traoré K., Rios E.F., Dubeux J.C., Boote K.J., Adesogan A. (2023). Performance of Food–Feed Maize and Cowpea Cultivars under Monoculture and Intercropping Systems: Grain Yield, Fodder Biomass, and Nutritive Value. Front. Anim. Sci..

[21] Belem A., Traoré M., Lankoande Y.F. (2024). Evaluation du Potentiel Fourrager des Résidus de Récoltes dans les Exploitations Cotonnières du Burkina Faso. Afr. J. Food Agric. Nutr. Dev..

[22] Mganga K.Z., Ndathi A.J.N., Wambua S.M., Bosma L., Kaindi E.M., Kioko T., Kadenyi N., Musyoki G.K., Van Steenbergen F., Musimba N.K.R. (2021). Forage Value of Vegetative Leaf and Stem Biomass Fractions of Selected Grasses Indigenous to African Rangelands. Anim. Prod. Sci..

[23] Ramirez R.G., Rusildi-González C., Hernandez-Piñeiro J.L., Maiti R. (1997). Nutritional Profile and Leaf Surface Structure of Some Monocotyledonous and Dicotyledonous Species for Grazing Ruminants in Semiarid Regions of Northeastern Mexico. J. Appl. Anim. Res..

[24] Niklas K.J. (1998). The Mechanical Roles of Clasping Leaf Sheaths: Evidence from *Arundinaria técta* (Poaceae) Shoots Subjected to Bending and Twisting Forces. Ann. Bot..

[25] Archimède H., Bastianelli D., Boval M., Tran G., Sauvant D. (2011). Ressources tropicales: Disponibilité et valeur alimentaire. INRA Prod. Anim..

[26] Sarnklong C., Cone J.W., Pellikaan W., Hendriks W.H. (2010). Utilization of Rice Straw and Different Treatments to Improve Its Feed Value for Ruminants: A Review. Asian-Australas. J. Anim. Sci..

[27] Etela I., Dung D.D. (2011). Utilization of Stover from Six Improved Dual-Purpose Groundnut (*Arachis hypogaea* L.) Cultivars by West African Dwarf Sheep. Afr. J. Food Agric. Nutr. Dev..

[28] Mero R.N., Udén P. (1998). Promising Tropical Grasses and Legumes as Feed Resources in Central Tanzania: VI. Nitrogen Balance in Growing Bulls Consuming Tropical Herbaceous Forage Legumes. Anim. Feed Sci. Technol..

[29] Minson D.J. (1991). Forage in Ruminant Nutrition.

[30] Cochran R.C., Galyean M.L. (1994). Measurement of In Vivo Forage Digestion by Ruminants. Forage Quality, Evaluation, and Utilization.

